# Mesenchymal Stem Cell‐Derived Mitochondria Enhance Extracellular Matrix‐Derived Grafts for the Repair of Nerve Defect

**DOI:** 10.1002/adhm.202302128

**Published:** 2023-11-16

**Authors:** Jun Bai, Bingbing Yu, Chaochao Li, Haofeng Cheng, Yanjun Guan, Zhiqi Ren, Tieyuan Zhang, Xiangyu Song, Zhibo Jia, Tianqi Su, Benzhang Tao, Haihao Gao, Boyao Yang, Lijing Liang, Xing Xiong, Xingyu Zhou, Lan Yin, Jiang Peng, Aijia Shang, Yu Wang

**Affiliations:** ^1^ Department of Neurosurgery General Hospital of Chinese People Liberty Army No. 28 Fuxing Road Beijing 100853 P. R. China; ^2^ Institute of Orthopedics The Fourth Medical Center of Chinese PLA General Hospital; Beijing Key Lab of Regenerative Medicine in Orthopedics Key Laboratory of Musculoskeletal Trauma and War Injuries PLA No. 51 Fucheng Road Beijing 100048 P. R. China; ^3^ Co‐innovation Center of Neuroregeneration Nantong University Nantong Jiangsu Province 226007 P. R. China; ^4^ Graduate School of Chinese PLA General Hospital No. 28 Fuxing Road Beijing 100853 P. R. China; ^5^ School of Materials Science and Engineering The Key Laboratory of Advanced Materials of Ministry of Education State Key Laboratory of New Ceramics and Fine Processing Center for Flexible Electronics Technology Tsinghua University Beijing 100084 P. R. China; ^6^ School of Medicine Nankai University Tianjin 300071 P. R. China; ^7^ School of Medicine Hebei North University Zhangjiakou 075051 P. R. China

**Keywords:** acellular nervous allograft, bioenergetics, mesenchymal stem cells, metabolism, mitochondrial transplantation, peripheral nerve regeneration

## Abstract

Peripheral nerve injuries (PNI) can lead to mitochondrial dysfunction and energy depletion within the affected microenvironment. The objective is to investigate the potential of transplanting mitochondria to reshape the neural regeneration microenvironment. High‐purity functional mitochondria with an intact structure are extracted from human umbilical cord‐derived mesenchymal stem cells (hUCMSCs) using the Dounce homogenization combined with ultracentrifugation. Results show that when hUCMSC‐derived mitochondria (hUCMSC‐Mitos) are cocultured with Schwann cells (SCs), they promote the proliferation, migration, and respiratory capacity of SCs. Acellular nerve allografts (ANAs) have shown promise in nerve regeneration, however, their therapeutic effect is not satisfactory enough. The incorporation of hUCMSC‐Mitos within ANAs has the potential to remodel the regenerative microenvironment. This approach demonstrates satisfactory outcomes in terms of tissue regeneration and functional recovery. Particularly, the use of metabolomics and bioenergetic profiling is used for the first time to analyze the energy metabolism microenvironment after PNI. This remodeling occurs through the enhancement of the tricarboxylic acid cycle and the regulation of associated metabolites, resulting in increased energy synthesis. Overall, the hUCMSC‐Mito‐loaded ANAs exhibit high functionality to promote nerve regeneration, providing a novel regenerative strategy based on improving energy metabolism for neural repair.

## Graphical abstract

Mitochondria from mesenchymal stem cells promote proliferation, migration, and respiratory metabolism of Schwann cells. Loading mitochondria onto decellularized nerve grafts promotes nerve regeneration and functional recovery. Mitochondrial transplantation therapy provides a novel regenerative strategy based on energy metabolism for nerve repair.

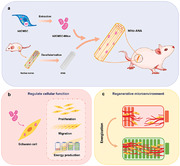

## 1 Introduction

Peripheral nerve injuries (PNI) account for ≈2.8% of all traumatic injuries, and affects over one million people globally each year.^[^ ^1^ ^]^ Nerve damage can occur due to compression, stretching, or complete transection.^[^ ^2^ ^]^ Among these, injuries that result in nerve transection and subsequent defects have the most severe consequences, often leading to significant motor and sensory impairments in the innervated area.^[^ ^3^ ^]^ Due to the presence of gaps and tension, end‐to‐end alignment of peripheral nerve defects during clinical surgical repair is often challenging. In such cases, suitable nerve grafts are selected to bridge the defect and guide nerve regeneration. Autologous nerve grafting remains the gold standard for the clinical treatment of PNI.^[^ ^4^ ^]^ However, autologous nerve transplantation is limited by several factors such as restricted donor availability, secondary surgical injury, and mismatch between donor and recipient nerves. Therefore, exploring alternative strategies for autologous nerve transplantation in peripheral nerve defect repair in clinical practice is urgent.^[^ ^5^ ^,^ ^6^ ^]^

The use of acellular nerve allografts (ANAs) as promising autologous nerve graft substitutes has been increasingly employed in the field of neural repair.^[^ ^7^, ^8^ ^]^ Compared to allogeneic nerves, ANAs effectively eliminate the immunogenicity associated with graft transplantation by removing cellular components from the native nerve. Simultaneously, the extracellular matrix integrity is preserved, ensuring a favorable microenvironment for nerve regeneration. This approach avoids immune rejection reactions associated with allogeneic nerve transplantation.^[^ ^9^, ^10^ ^]^ However, current research has shown that the therapeutic efficacy of using ANAs alone for nerve defect repair still needs improvement.^[^ ^11^ ^]^ Mesenchymal stem cells (MSCs)—with anti‐inflammatory, antiapoptotic, immunomodulatory, and angiogenic properties, as well as ease of isolation and expansion—are often used as excellent additions to enhance the reparative effects of ANAs.^[^ ^12^, ^13^ ^]^ Traditionally, it was believed that MSCs exert their effects mainly through proliferation and differentiation to replace functionally compromised cells at the site of injury. However, increasing evidence has suggested that the survival rate and efficiency of MSCs in differentiating into target cells after transplantation are generally too low to match the observed beneficial reparative effects.^[^ ^14^ ^]^ Recent and compelling evidence now suggests that stem cells exert their effects indirectly through the paracrine release of cell‐active factors (e.g., growth factors)^[^ ^15^ ^]^ and vesicles (e.g., exosomes)^[^ ^16^ ^]^ in tissue repair.^[^ ^17^ ^]^ The advancement of “cell‐free therapies” derived from stem cells has emerged as a promising approach, providing reparative effects comparable to stem cell therapies. These therapies offer the advantage of avoiding the biological safety concerns associated with direct stem cell transplantation.^[^ ^18^ ^]^ Furthermore, the utilization of cell‐secreted components presents additional benefits, including convenient storage and stable bioactivity.^[^ ^19^ ^]^

Mitochondria are key organelles that determine cellular energy and viability and produce adenosine triphosphate (ATP) through oxidative phosphorylation.^[^ ^20^ ^]^ In addition to producing energy, mitochondria are involved in fatty acid biosynthesis and play a key regulatory role in senescence, immune response, cell survival, and autophagy.^[^ ^21^ ^]^ After tissue injury, the disruption of the mitochondria at the site of injury can lead to impaired ATP synthesis, increased reactive oxygen species, and calcium overload, a complex postinjury pathological microenvironment that hinders neural regeneration.^[^ ^22^ ^]^ Because of the important role of the mitochondria in maintaining cellular physiological functions, considerable attention has been focused on the regulation of mitochondrial function, bringing up the concept of “mitochondrial medicine.”^[^ ^23^ ^]^ Current strategies to regulate mitochondrial function usually involve the addition of mitochondria‐based uncoupling agents, metabolic modulators, or antioxidants.^[^ ^24^, ^25^ ^]^ As the mitochondrial exchange phenomenon occurring spontaneously within the organism is increasingly being captured, it has been observed that when tissues or cells are damaged, the mitochondria can be spontaneously transferred between cells to rescue the function of the damaged site.^[^ ^26^, ^27^, ^28^ ^]^ For example, astrocytes in mice release functional mitochondria into neurons and exert neuroprotective effects after stroke.^[^ ^29^ ^]^ Based on this pathophysiological phenomenon, a new “cell‐free therapy” strategy was introduced, namely, “mitochondrial transplantation,” in which exogenous healthy mitochondria are directly transplanted into the injured microenvironment and internalized by the cells in the injured area, replacing the damaged mitochondria in the cells. This strategy promotes tissue regeneration by improving the pathological microenvironment of the injury.^[^ ^30^ ^]^ However, while current research on mitochondrial transplantation has mainly focused on myocardial ischemia‐reperfusion,^[^ ^31^ ^]^ comprehensive studies on the nervous system, especially in PNI, are largely scarce. It is well‐established that the nervous system uses huge energy to maintain normal physiological activity, accounting for ≈20% of the body's energy consumption.^[^ ^32^ ^]^ After nerve injury, mitochondrial dysfunction accompanied by energy depletion at the site of injury hinders nerve repair. Studies have shown that higher numbers and motility of mitochondria in damaged cells may provide better regenerative capacity in the peripheral and central nervous systems,^[^ ^33^ ^]^ making mitochondria a key therapeutic target. Based on the above research and theoretical basis, we hypothesize that it is possible to directly replenish damaged peripheral nerves with “functionally healthy” mitochondria to promote nerve regeneration by “energizing” the site of injury.

**Scheme 1:**
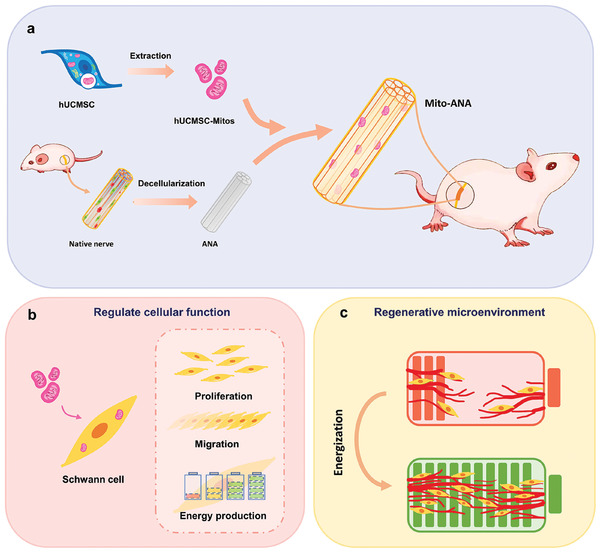
Schematic diagram of MSC‐derived mitochondria‐enhanced extracellular matrix‐derived grafts for the repair of peripheral nerve defects. a) Extraction of hUCMSC‐Mitos and preparation of ANA, Mito‐ANA for the repair of sciatic nerve defects. b) Functional modulation of SCs by hUCMSC‐Mitos. c) Mito‐ANA reshapes the regenerative microenvironment by “energizing” the injury site.

Therefore, we developed an energy‐empowering strategy for nerve repair through mitochondrial transplantation. Specifically, we constructed a cell‐derived extracellular matrix‐based graft loaded with human umbilical cord‐derived mesenchymal stem cells‐derived mitochondria (hUCMSC‐Mitos) and explored its therapeutic potential in nerve regeneration. hUCMSC‐Mitos were prepared and purified using the Dounce homogenization method combined with low‐temperature ultracentrifugation. In in vitro experiments, we explored the functional regulation of mitochondrial transplantation on the proliferation and migration capabilities of cultured Schwann cells (SCs) and assessed the bioenergetic characteristics of SCs after transplantation of mitochondria using a Seahorse XF96 Analyzer. To track changes in gene expression, we analyzed total RNA profiles of SCs under exogenous mitochondrial regulation. ANAs that retained the natural 3D primitive structure of the nerve were used to load functional mitochondria, and the constructed hUCMSC‐Mito‐loaded ANA graft (Mito‐ANA) was used to bridge a 10 mm sciatic nerve defect. The evaluation of the repair process involved the assessment of pathological, behavioral, and functional indicators. To gain insights into the changes occurring in energy production capacity, metabolic products, and pathways within the injured area following nerve damage, metabolomics and bioenergetic profiling were employed as analytical tools. This study offers a novel research strategy for nerve regeneration based on improving the energy and metabolism of the damaged microenvironment (**Scheme** **1**).

## 2 Results

### 2.1 Extraction and Biological Characteristics of hUCMSC‐Mitos

**Figure 1:**
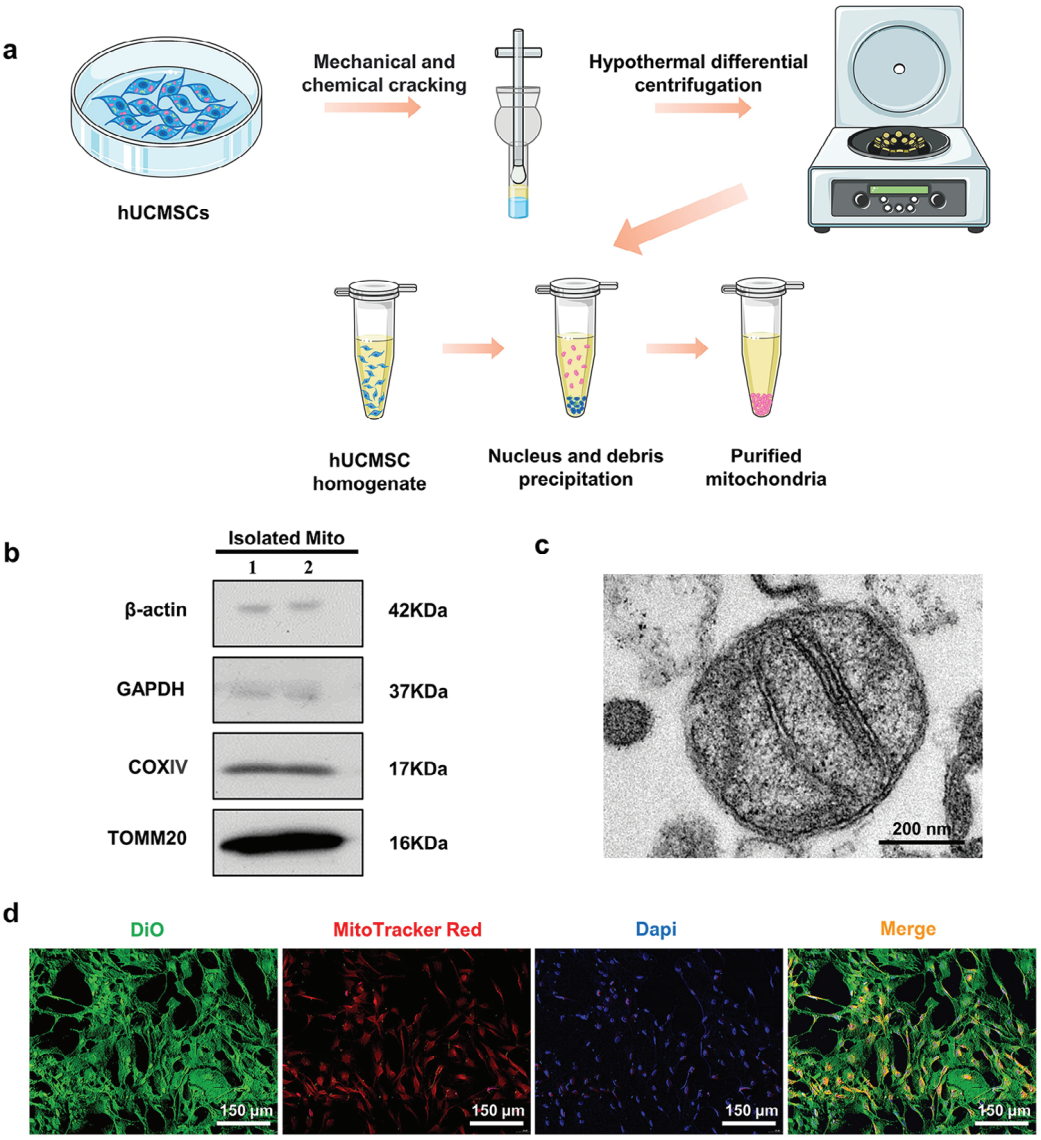
Preparation, identification, and uptake of hUCMSC‐Mitos. a) Workflow for the extraction of hUCMSC‐Mitos. b) Quantification of mitochondrial‐specific markers by Western blot (mitochondrial specific marker: COX IV and TOMM20; cytoplasmic marker proteins: β‐actin and GAPDH). c) TEM image of hUCMSC‐Mitos. d) Uptake of hUCMSC‐Mitos by SCs (SCs' cell membrane: DiO; hUCMSC‐Mitos: MitoTracker Red; Nucleus: DAPI).

Mitochondrial function is impaired during high passaged MSC or MSC senescence,^[^ ^34^, ^35^ ^]^ we examined the status of mitochondria in the cells, and found that the P3 generation hUCMSCs had lower ROS levels and higher levels of ATP production compared to the P8 generation hUCMSCs (Figure S1, Supporting Information), which represent a higher quality of mitochondrial function, so we used P3 generation hUCMSCs as mitochondrial donor cells. We obtained functional mitochondria from hUCMSCs using a combination of mechanical and chemical methods to lyse cells, followed by multiple differential centrifugation steps at low temperatures to separate the mitochondria from the cellular homogenate (**Figure** **1**a). The purity of the isolated mitochondria was validated through Western blot analysis. Mitochondrial marker proteins cytochrome c oxidase subunit IV (COX IV) and translocase of the outer mitochondrial membrane complex subunit 20 (TOMM20) were highly abundant in the isolated mitochondria, while cytoplasmic marker proteins β‐actin and GAPDH were only present at low levels. The results indicated that the extracted mitochondria had a high purity (Figure 1b). Subsequently, we evaluated the structural characteristics of the isolated mitochondria. Under transmission electron microscopy (TEM), hUCMSC‐Mitos displayed well‐defined structures with sizes ranging from 0.1 to 1 µm. The mitochondria exhibited various shapes, including circular, elongated, and rod‐like structures. Mitochondrial membranes were intact without signs of wrinkling, and clear mitochondrial cristae were observed, indicating the integrity of the isolated mitochondria (Figure 1c). We examined the activity of extracellular mitochondria and found that ATP production capacity of mitochondria decreased significantly after 12 h of detachment from the cell, and extracellular mitochondria basically lost the ability to produce ATP at 48 h (Figure S2, Supporting Information). The protein concentration of extracted mitochondria was measured using the bicinchoninic acid (BCA) method, and a value of 0.177 mg mL^−1^ was obtained. These experimental results demonstrated that highly pure and structurally intact “functional” mitochondria were extracted from hUCMSCs.

SCs are important glial cells in the peripheral nervous and assist nerve repair through myelination.^[^ ^36^ ^]^ Therefore, we evaluated the functional impact of hUCMSC‐Mitos on SCs to uncover the potential mechanisms by which hUCMSC‐Mitos promote nerve regeneration. To determine whether SCs could internalize hUCMSC‐Mitos, SC membranes were labeled with DiO, and hUCMSC‐Mitos were labeled with MitoTracker Red for tracking purposes. Cocultures of hUCMSC‐Mitos and SCs were established. Using fluorescence microscopy, it was found that SC membranes with green fluorescence contained numerous exogenous mitochondria labeled with red fluorescence (Figure 1d). We also observed the survival of intracellular mitochondria at multiple time points, where red fluorescence of mitochondria could be observed in SCs on days 1 and 3, and red fluorescence became faint and difficult to observe at day 5 (Figure S3, Supporting Information). High‐content analysis system captured the dynamic process of SCs internalizing hUCMSC‐Mitos. After 10 min of coculture, red fluorescence signals started to appear in the cytoplasm of SCs. With prolonged coculture time, the mitochondria continued to accumulate in the cytoplasm. After 8 h, hUCMSC‐Mitos were observed to be localized within the cytoplasm of SCs, and 100% of SCs successfully internalized hUCMSC‐Mitos (Movie S1, Supporting Information). Collectively, these experimental results demonstrate that SCs have a rapid uptake capacity for hUCMSC‐Mitos.

### 2.2 Effects of hUCMSC‐Mitos on Proliferation, Migration, and Bioenergy Spectrum of SCs

**Figure 2:**
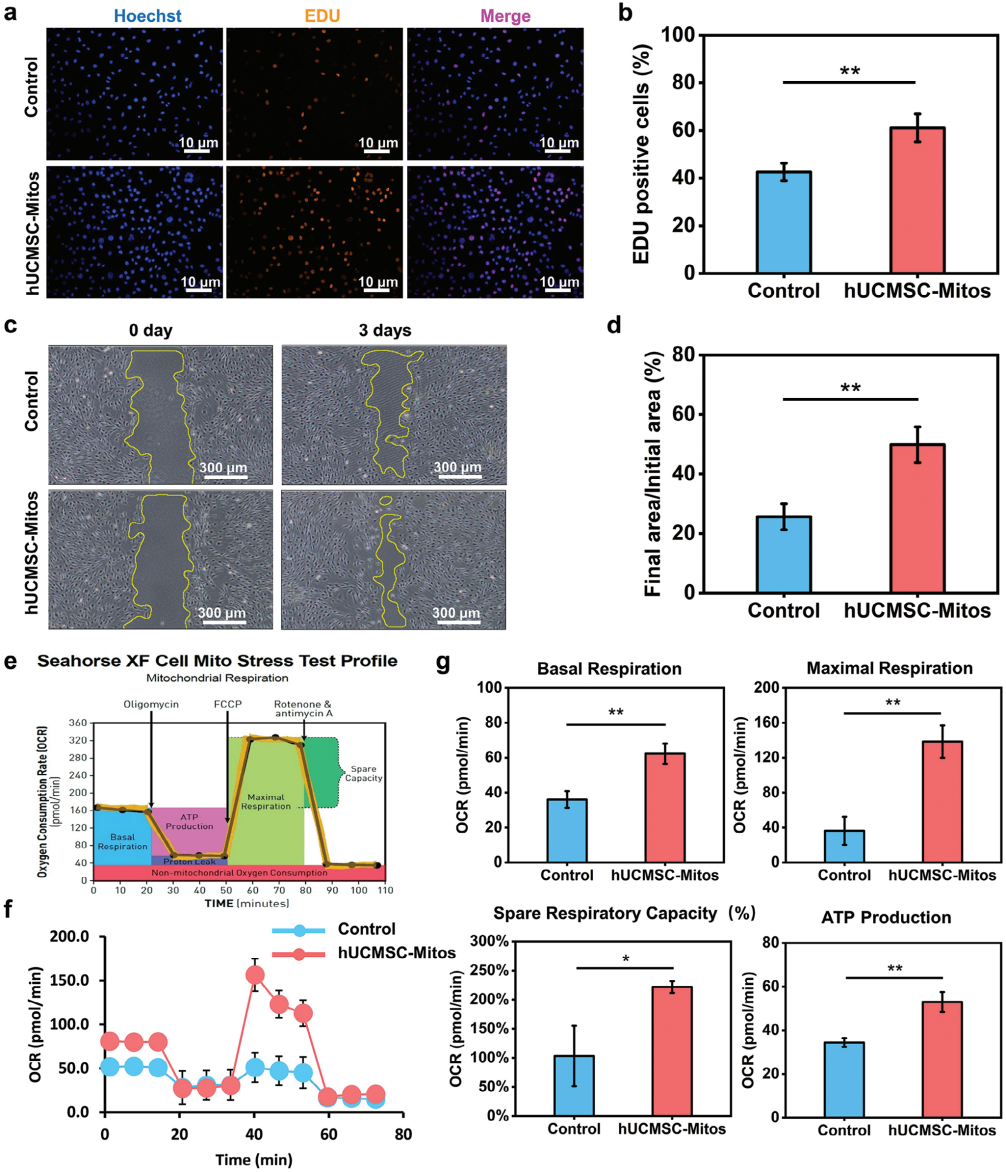
SCs behavior with the modulation of hUCMSC‐Mitos. a) EdU staining images of SCs, with orange representing newly proliferated cells and blue representing all cells. b) Ratio of double‐stained cells (EdU‐positive) to the total number of cells. c) Representative images of scratch assay at the initial and final time points. d) Quantification the migration area percentage of SCs. e) Schematic diagram of Seahorse XF Cell Mito Stress Test parameters. f) Seahorse analysis of cellular bioenergetics profile, namely, oxygen consumption rate (OCR, pmols O_2_/min) throughout the assay. g) Assessment of basal respiration, maximal respiration, spare respiratory capacity, and ATP production. Significantly different (one‐way analysis of variance (ANOVA)): *p < 0.05 and **p <0.01.

We further investigated the effects of hUCMSC‐Mitos on various cellular functions of SCs. First, we compared the cell viability among the untreated group, low‐concentration hUCMSC‐Mito‐treated group (5 µg mL^−1^), and high‐concentration hUCMSC‐Mito‐treated group (20 µg mL^−1^) using the Cell Counting Kit‐8 (CCK‐8) cell proliferation assay. After 1 day of hUCMSC‐Mito internalization in SCs, there was no statistical significance in absorbance between the control group (0.368 ± 0.011) and the low‐concentration group (0.378 ± 0.017), while the absorbance in the high‐concentration group (0.425 ± 0.012) was higher than these two groups. At day 3, the absorbance in the high‐concentration group (1.72 ± 0.074) was still significantly higher than the untreated group (1.117 ± 0.094) and the low‐concentration group (1.322 ± 0.065) (Figure S4, Supporting Information). The SC proliferation of the two hUCMSC‐Mito treatment groups was higher than that of the control group, with the high‐concentration group exhibiting a more significant proliferative effect that the low‐concentration group. The proliferative‐promoting effect of hUCMSC‐Mito‐treated SCs was more evident on day 3, indicating a dose‐dependent and time‐dependent effect of mitochondrial transplantation on SC proliferation. Based on these results, a concentration of 20 µg mL^−1^ of hUCMSC‐Mitos was utilized for subsequent experiments, and the third day after hUCMSC‐Mitos addition was the time point for transcriptomic sequencing and energy metabolism detection. Further, the 5‐ethynyl‐2′‐deoxyuridine (EdU) cell proliferation assay was applied to compare the proliferative capacity of the hUCMSC‐Mito‐treated group with the untreated group. Cells stained with both EdU (orange) and Hoechst (blue) were considered newly proliferated cells, and the hUCMSC‐Mito group exhibited a higher proportion of double‐stained cells than the control group (**Figure** **2**a). EdU‐positive rates were 61.1 ± 3.7% and 42.6 ± 5.9% in hUCMSC‐Mito and control groups, respectively (Figure 2b), indicating an enhanced proliferative capacity of SCs after 3 days of hUCMSC‐Mito treatment.

The high motility of SCs is a pivotal factor in peripheral nerve regeneration.^[^ ^37^ ^]^ The migration capacity of SCs after mitochondrial transplantation was investigated using a wound‐healing assay. Compared with the control group, the hUCMSC‐Mito group exhibited enhanced migration ability after 3 days of hUCMSC‐Mito treatment, with faster closure of the scratch gap between cells (Figure 2c). The migration area ratio of the hUCMSC‐Mito group was dramatically increased compared with that of the control group (25.6 ± 4.4 vs 49.9 ± 6%) (Figure 2d). This data indicated that hUCMSC‐Mitos enhance the motility of SCs.

We further examined the bioenergetics profile of SCs after mitochondrial transplantation. A Seahorse XF96 Analyzer was used to measure the respiration of SCs after mitochondrial transplantation. The oxygen consumption rate (OCR) under no‐intervention conditions and after sequential addition of oligomycin, carbonyl cyanide 4‐(trifluoromethoxy)phenylhydrazone (FCCP), and rotenone/antimycin A (Rot/AA) was measured. The results obtained, including basal respiration, maximal respiration, spare respiratory capacity, and ATP production, were calculated by subtracting nonmitochondrial oxygen consumption (Figure 2e). After 3 days of mitochondrial transplantation, the hUCMSC‐Mito group exhibited increased overall cellular oxidative phosphorylation levels and a higher total OCR than the control group (Figure 2f). Specifically, the hUCMSC‐Mito group showed increased basal respiration, indicating the enhanced respiratory capacity in the resting state. Maximal respiration and spare respiratory capacity were increased, suggesting enhanced respiratory potential and capacity under stress conditions in SCs. The ATP production capacity was significantly increased in the hUCMSC‐Mito group (Figure 2g). Taken together, these results suggested that mitochondrial transplantation with hUCMSC‐Mitos enhances the aerobic respiration level and mitochondrial ATP production capacity of SCs.

### 2.3 RNA‐Sequencing (RNA‐seq) Data Analysis

**Figure 3:**
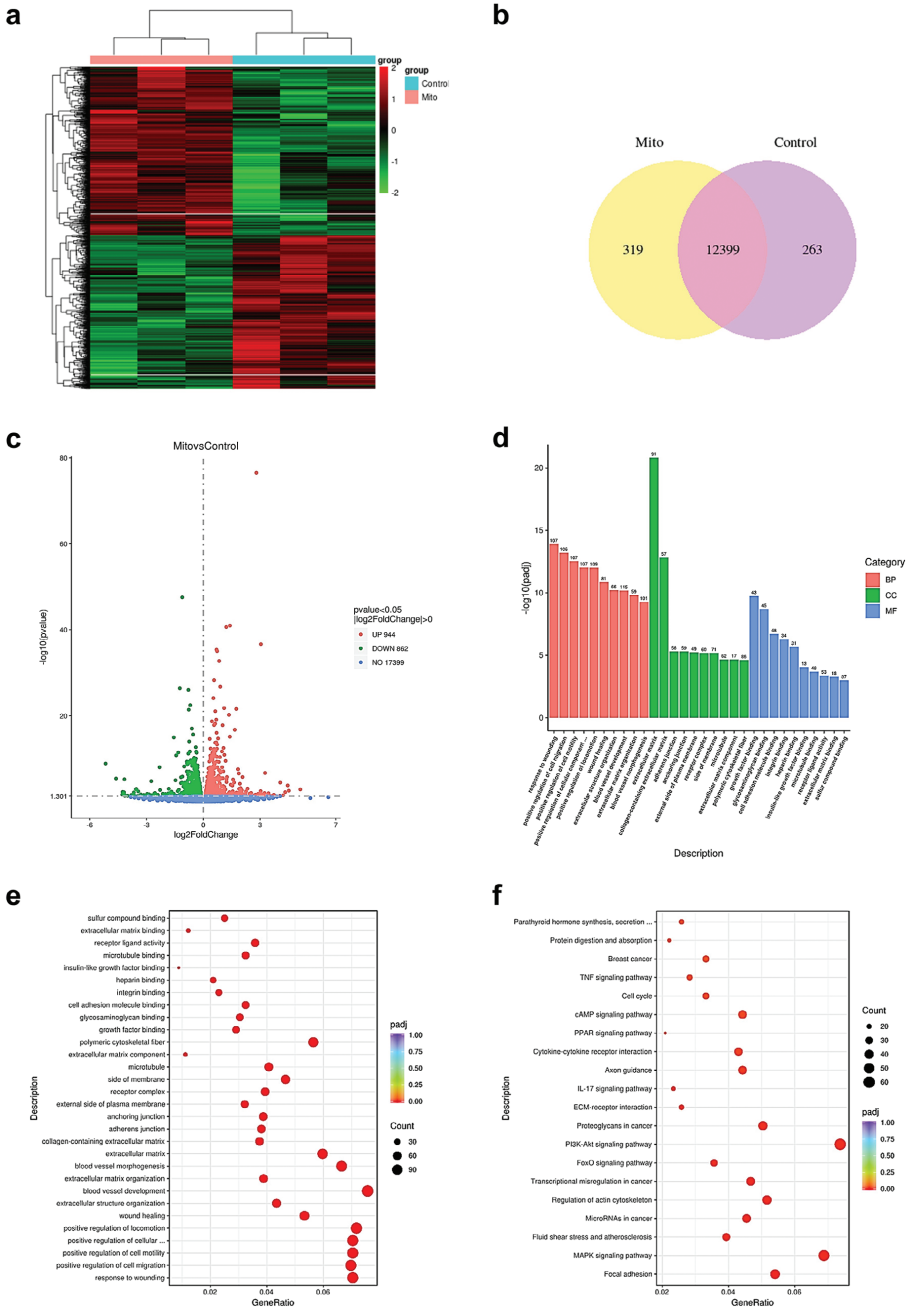
RNA‐seq analysis of SCs treated with hUCMSC‐Mitos at day 3. a) Heatmap displaying clustering of differentially expressed genes. Mito represents the hUCMSC‐Mitos group, with green indicating low expression and red indicating high expression. b) Venn diagram showing the overlap and unique expression of genes between the hUCMSC‐Mitos group and control group. c) Volcano plot displaying the distribution of differentially expressed genes between the hUCMSC‐Mitos group and control group. Red dots represent upregulated genes, and green dots represent downregulated genes. d) Gene ontology (GO) analysis of differentially expressed genes between the hUCMSC‐Mitos group and control group (BP: Biological Processes; CC: Cellular Components; MF: Molecular Functions). e) Scatter plot displaying the top 30 significantly enriched GO terms. f) Scatter plot displaying the top 20 significantly enriched KEGG pathways.

We conducted whole transcriptome sequencing to explore the expression of differential and important genes in SCs after mitochondrial transplantation. Previous studies indicated significant functional changes in SCs at 3 days. Therefore, we selected 3 days after mitochondrial transplantation as the time point for RNA‐seq data analysis. Comparative analysis of differentially expressed genes (DEGs) between hUCMSC‐Mito and control groups revealed 944 upregulated and 862 downregulated genes (**Figure** **3**c). The Venn diagram showed that at 3 days, there were 319 DEGs in the hUCMSC‐Mito group and 263 DEGs in the control group (Figure 3b). A gene clustering heatmap was used to visualize the distribution of DEGs between the two groups (Figure 3a). Gene ontology (GO) enrichment analysis showed that DEGs were enriched in three modules, including biological process (BP), cellular component (CC), and molecular function (MF) modules. For the BP module, upregulated genes were significantly enriched in positive regulation of cell migration and motility, response to wounding, blood vessel development, and blood vessel morphogenesis. For the CC module, the DEGs were enriched in the extracellular matrix, collagen‐containing extracellular matrix, and microtubule. For the MF module, DEGs were involved in growth factor binding, glycosaminoglycan binding, integrin binding, insulin‐like growth factor binding, and extracellular matrix binding (Figure 3d,e). Furthermore, Kyoto Encyclopedia of Genes and Genomes (KEGG) pathway analysis revealed that DEGs were enriched in pathways such as the PI3K‐Akt signaling pathway, MAPK signaling pathway, focal adhesion, regulation of actin cytoskeleton, cytokine–cytokine receptor interaction, axon guidance, cAMP signaling pathway, and cell cycle (Figure 3f).

### 2.4 Characteristics of Mito‐ANAs

Freshly harvested sciatic nerves underwent a series of processes, including immersion in a hypotonic solution, low‐temperature treatment, and chemical decellularization, for ANA preparation. The efficiency of cellular removal and structural preservation of ANA was validated. The natural nerve tissue contains cellular components and has a high DNA content. After decellularization, the DNA content significantly decreased (Figure S5, Supporting Information). Immunofluorescence staining was performed to detect antigens in fresh nerves and decellularized nerve grafts. The specific markers for axons (NF‐200) and SCs (S‐100β) were positively expressed in the natural nerve tissue, indicating the presence of allogeneic axons and SC antigens in the graft. However, the ANA showed negative staining for NF‐200 and S‐100β, indicating the complete removal of axonal and SC antigens from the ANA (Figure S6, Supporting Information).

**Figure 4:**
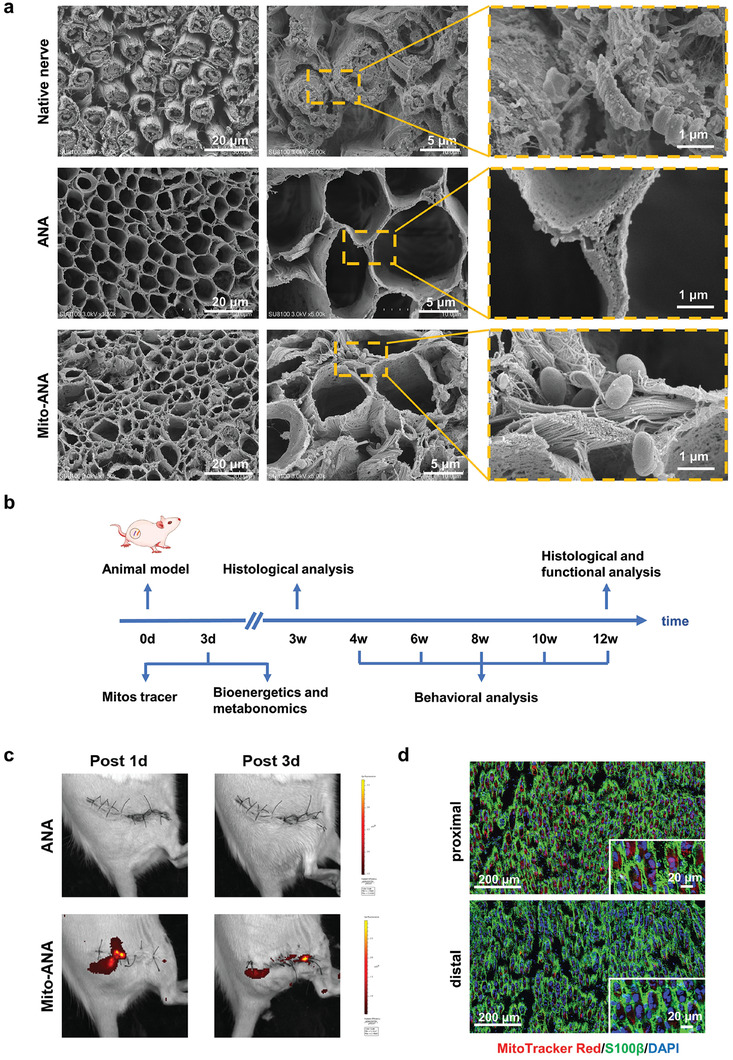
Construction of ANA and hUCMSC‐Mitos grafts for peripheral nerve regeneration. a) SEM images of native nerve, ANA, and Mito‐ANA. b) Schematic diagram of the evaluation of nerve grafts at key time points after injury. c) In vivo images captured by the IVIS imaging system, showing fluorescence signals emitted by hUCMSC‐Mitos in the Mito‐ANA group at day 1 and day 3 postsurgery compared to the ANA group. d) Immunofluorescence staining showing the uptake of hUCMSC‐Mitos by SCs in the proximal (upper) and distal (lower) nerve stumps on the 3rd day after surgery. (SCs: S100β; hUCMSC‐Mitos: MitoTracker Red; Nucleus: DAPI).

We used a scanning electron microscope to observe the surface and interior structure of the three types of grafts. The natural sciatic nerve was covered with cellular and antigenic components, resulting in a rough surface. However, the ANA preserved the original 3D physiological structure of the natural nerve tissue, which was advantageous for guiding axonal regeneration. The surface of ANA exhibited a reticular porous structure, which ensured the transport of oxygen and loaded components to support cell migration and proliferation. After loading the mitochondria onto ANA, structurally intact mitochondria were uniformly distributed on the ANA surface. Overall, these results indicated that an immunologically inert and energy‐supplying neural graft was successfully constructed by chemical decellularization and mitochondrial loading (**Figure** **4**a). We performed routine blood tests at 3 days after implantation, focusing on lymphocyte, leukocyte, monocyte, eosinophil, and neutrophil counts, and no inflammatory or immune abnormalities were detected after implantation (Figure S7, Supporting Information).

After the successful construction of the graft, we implanted it into the sciatic nerve defect in rats and observed whether Mito‐ANA could accelerate nerve regeneration. The key detection time points in the experiment are listed (Figure 4b). We tracked the distribution of hUCMSC‐Mitos in the body by monitoring the MitoTracker Red fluorescence signal of the neural graft. In vivo fluorescence imaging of animals revealed that at day 1, the mitochondrial fluorescence signal in Mito‐ANA was continuously distributed along the course of the graft. By day 3, the mitochondrial fluorescence signal accumulated near the proximal and distal ends of the transected sciatic nerve, with a larger dispersion area in the proximal end. This suggested that the mitochondria within the graft were taken up by the damaged nerve tissue at the transection site (Figure 4c). After 3 days of transplantation, we performed pathological staining on the two‐terminal tissue of the injured nerves. We observed red fluorescent‐labeled hUCMSC‐Mitos within the SCs of the nerve tissue, with a larger fluorescent area and stronger signal intensity in the proximal end compared with the distal end. This implied a higher uptake rate of mitochondria in SCs at the proximal end, possibly due to the higher metabolic activity of SCs in the damaged nerve at the proximal end.^[^ ^38^ ^]^ Based on this, it can be inferred that hUCMSC‐Mitos play a key regulatory role in the function of SCs during nerve regeneration (Figure 4d). Therefore, our subsequent energy metabolism analysis focused on the proximal end of the injury site, which exhibited higher mitochondrial uptake.

### 2.5 Remodeling of an Energy Metabolism Microenvironment

To explore the variation of Mito‐ANA transplantation on the metabolic profile at the injury site, we performed metabolomic analysis on neural tissues from ANA and Mito‐ANA groups three days after surgery. Sixteen samples (8 samples per group) were analyzed for the expression levels of metabolites. Partial least‐squares discriminant analysis models were established for each group, and model evaluation parameters (R2 and Q2) were obtained through sevenfold cross‐validation. The values of R2 and Q2 were close to 1, indicating that the models were stable and reliable. We also conducted tests to check for “overfitting” of the models, and it was confirmed that the models were sound.

**Figure 5:**
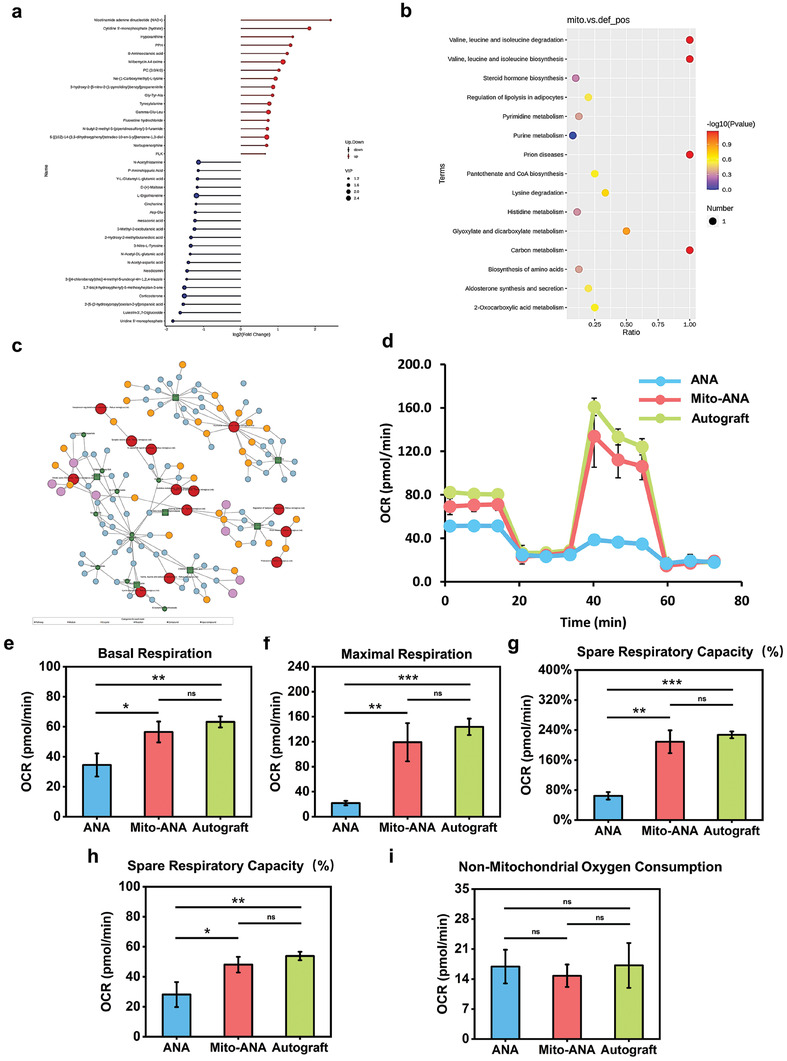
Energy metabolism analysis of the injured microenvironment three days after graft transplantation. a) Bar plot showing differential metabolites between the Mito‐ANA group and the ANA group. Red represents upregulated metabolites, and blue represents downregulated metabolites. b) Scatter plot displaying significantly enriched KEGG pathways of metabolites. c) KEGG regulatory network diagram integrating all pathway nodes, including metabolites, reactions, enzymes, modules, and pathways. d) Bioenergetic profiling of the proximal injury site, namely, oxygen consumption rate (OCR, pmols O_2_/min) throughout the assay. (e–i) Assessment of basal respiration, maximal respiration, ATP production, spare respiratory capacity, and nonmitochondrial respiration. Significantly different (one‐way analysis of variance (ANOVA)): ns, not significant, *p < 0.05, **p <0.01, and ***p < 0.001.

Based on significantly different metabolites in each group, we created a lollipop chart to identify upregulated metabolites in the Mito‐ANA group compared with the ANA group. These metabolites included NAD+, 5′‐phosphoribosylcytidine (hydrate), xanthine, PPH, 8‐aminooctanoic acid, Gly‐Tyr‐Ala, and tyrosine‐proline (**Figure** **5**a). Among them, NAD+ plays a crucial role in cellular respiration and mitochondria, participating in redox reactions in the three major energy metabolism pathways, including glycolysis, the citric acid cycle, and oxidative phosphorylation. NAD+ accepts electrons and negative charges in these metabolic pathways, which convert it to its reduced form, NADH, which ultimately converts energy into ATP through the electron transport chain. Consequently, we identified significantly enriched KEGG pathways associated with these metabolites and calculated their topological impact. For the significantly enriched KEGG pathways, we created a metabolic pathway map. The results showed differences in amino acid biosynthesis, carbon metabolism, pyruvate and citrate metabolism, pantothenate and CoA biosynthesis, and 2‐oxocarboxylic acid metabolism between control and Mito‐ANA groups (Figure 5b). In the enriched network, we visualized all pathway nodes (including metabolites, reactions, enzymes, modules, and pathways) as a KEGG regulatory network diagram, revealing the crucial role of the tricarboxylic acid (TCA) cycle‐related metabolic network (Figure 5c). The TCA cycle is a shared pathway in carbohydrate, lipid, and amino acid metabolism, playing an important role in regulating cellular energy and fate.^[^ ^39^ ^]^ Our data suggested that postinjury metabolic disorders can be reversed by the transplantation of Mito‐ANA, possibly by upregulating NAD+ to enhance ATP production via the TCA cycle and reshaping the metabolic microenvironment.

Seahorse XF96 analysis was performed to evaluate the mitochondrial respiration parameters in the injured proximal nerve tissue. The observed value suggested that the autograft group had the highest mitochondrial respiration OCR after 3 days of sciatic nerve transection. The ANA group exhibited the lowest OCR level after 3 days of sciatic nerve transection, while the Mito‐ANA transplantation group showed no significant difference compared with the autograft group (Figure 5d). This indicated that compared with the autograft group, a pure ANA repair of the sciatic nerve defect exhibited a low level of OCR, while Mito‐ANA achieved energy production levels similar to those of the autograft group. When comparing the OCR values among the three groups, the ANA group had lower levels of basal respiration, maximal respiration, spare respiratory capacity, and ATP production OCR after 3 days of sciatic nerve transection, suggesting a lower respiratory capacity in the ANA group (Figure 5e–g). The Mito‐ANA group showed significantly improved respiratory capacities compared with the ANA group, which was similar to the autograft group (Figure 5h). The detection of nonmitochondrial respiration showed no significant differences among the three groups (Figure 5i). These findings confirmed that during nerve regeneration, ANA alone cannot restore the respiratory levels at the injury site, while the use of Mito‐ANA can generate respiratory capacities similar to those of the autograft.

Through metabolomics analysis and Seahorse detection, we described the metabolic changes during nerve regeneration, revealing metabolic disorders and energy depletion caused by alterations in metabolites and impaired tissue respiration after sciatic nerve injury. By supplementing exogenous hUCMSC‐Mitos, we reshaped the energy metabolic microenvironment during the process of nerve regeneration.

### 2.6 Effects of Mito‐ANA on Nerve Regeneration

**Figure 6:**
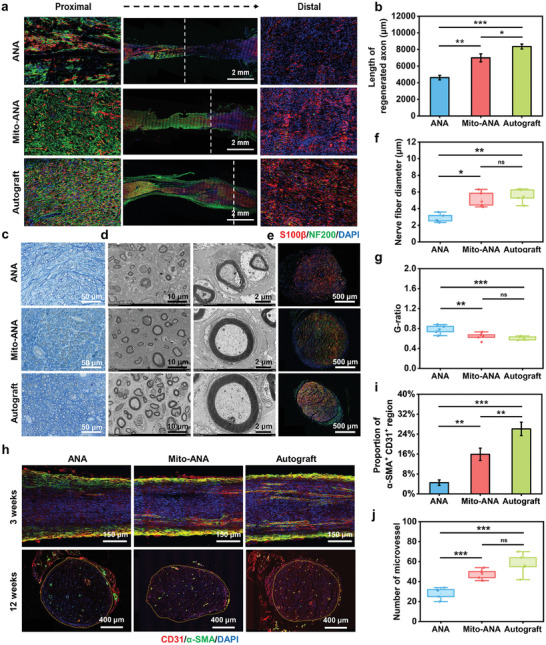
Histological evaluation of sciatic nerve repair in vivo. a) Images of longitudinal sections stained by immunofluorescence at 3 weeks post‐transplantation; the white dashed line represents the proximal regenerating length of axons, with the distal and magnified images shown on both sides (SCs: S100‐β; Axons: NF200; DNA: DAPI). b) Statistical analysis of axonal regrowth lengths in each group. c) Toluidine blue staining of transverse sections of the nerves at 12 weeks. d) TEM image of transverse sections of the regenerated sciatic nerve at 12 weeks. e) Immunofluorescence images of transverse sections in the middle segment of the nerve at 12 weeks. f) Average diameters of myelinated axons. g) Average area‐based g‐ratio. h) Representative immunofluorescence staining images of nerve transplants for CD31 and α‐SMA (smooth muscle: α‐SMA; endothelial cells: CD31; DNA: DAPI). i) Area ratio of α‐SMA+ CD31+ costaining area in the proximal section. j) Number of microvessels in the midsection of the nerve. *n* = 5 independent animals per group taken at week 12. Significantly different (one‐way analysis of variance (ANOVA)): ns, not significant, *p < 0.05, **p <0.01, and ***p < 0.001.

To investigate the ability of Mito‐ANAs to promote early nerve regeneration, the regenerative lengths of the sciatic nerve were evaluated three weeks after surgery. Immunofluorescent staining for S100β and NF200 was performed on longitudinally sectioned sciatic nerves. The dyed image results showed that the regenerative lengths of the nerves in the Mito‐ANA group were significantly longer than those of the ANA group. High‐magnification imaging of the proximal and distal ends revealed densely packed and orderly arranged red‐green double‐stained regions with a high fluorescence density. The autograft group exhibited the highest nerve fiber density represented by S100β and NF200, with the most compact and orderly arrangement (**Figure** **6**a). Autograft significantly enhanced rapid axonal extension and movement of SCs from the proximal and distal ends, resulting in a regenerated axonal length of 8356 ± 302 µm. The Mito‐ANA group followed with a regenerated axonal length of 6994 ± 477.6 µm, while the ANA group showed the lowest level of axonal regeneration, with a nerve regenerative length of 4616 ± 269.5 µm (Figure 6b).

Pathological sectioning, staining, and imaging were performed on the rat nerves at 12 weeks postsurgery. Toluidine blue staining was used to observe the density of regenerating axons. The axonal density in autograft and Mito‐ANA groups was higher than that in the ANA group (Figure 6c). The structural characteristics of regenerating nerve fibers were examined under electron microscopy (Figure 6d), and the diameter of nerve fibers and G‐ratio were quantified. The nerve fiber diameter was the highest in the autograft group (5.539 ± 0.818 µm), followed by the Mito‐ANA group (5.133 ± 0.911 µm) and the ANA group (2.85 ± 0.514 µm) (Figure 6f). The G‐ratio was highest in the ANA group (0.78 ± 0.087), while there was no significant difference between the Mito‐ANA group (0.643 ± 0.074) and the autograft group (0.607 ± 0.036) (Figure 6g). These results suggested that Mito‐ANAs play a promoting role in nerve regeneration and the formation of myelin sheaths. Additionally, S100β and NF200 staining of transverse sections of the sciatic nerve revealed a higher density of regenerated axons and a better myelin sheath quality in the Mito‐ANA conduit group, while the ANA transplantation group exhibited only a small number of SCs and regenerating axons (Figure 6e).

In terms of early angiogenesis, we performed longitudinal sectioning of the proximal sciatic nerve at 3 weeks postsurgery and conducted CD31 and α‐SMA staining. Mito‐ANAs significantly supported the growth of regenerating blood vessels in the proximal region, with the long axis of the blood vessels generally aligned with the direction of nerve regeneration. The extent of vascular regeneration was observably higher in the Mito‐ANA group compared with the ANA group. Autograft had the highest level of recovery (Figure 6h,i). At 12 weeks postsurgery, immunofluorescent staining was performed on transverse sections of the sciatic nerve (Figure 6h). The number of microvessels formed was higher in both the autograft group (57.4 ± 10.57) and the Mito‐ANA group (47.4 ± 5.079) than in the ANA group (28.4 ± 5.77) (Figure 6j). After bridging with Mito‐ANAs, early microvessel generation was promoted, providing a pathway for the delivery of oxygen and nutrients during the regeneration process.

Collectively these results indicate that Mito‐ANA transplantation enhances its supportive role in peripheral nerve defect repair by promoting axonal regeneration, myelin sheath formation, and microvessel generation. Significant promotion of nerve regeneration was confirmed in both the early (3 weeks) and late (12 weeks) stages.

### 2.7 Effects of Mito‐ANA on Functional Recovery

**Figure 7:**
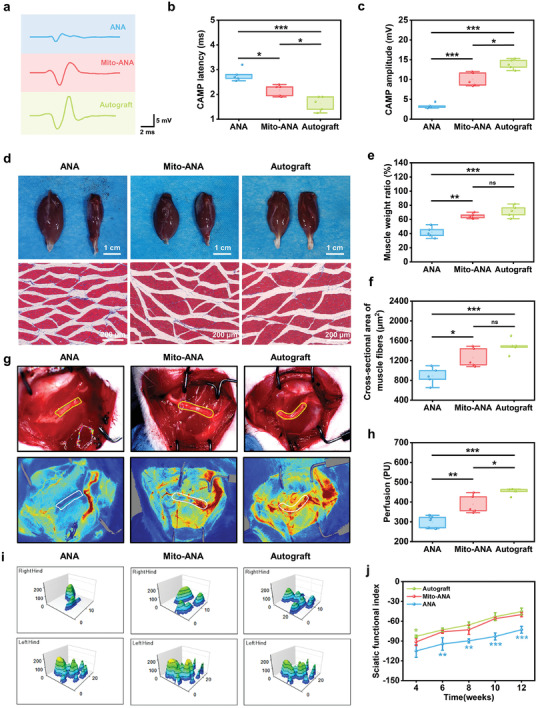
Behavioral and functional assessment of sciatic nerve repair. a) Representative compound muscle action potentials (cMAPs) of the injured side. b) Statistical analysis of cMAP latency at the injured side. c) Statistical analysis of cMAP amplitude at the injured side. d) Gross images and Masson's trichrome staining images of the gastrocnemius muscles in each group. e) Statistical analysis of the wet weight ratio of the gastrocnemius muscles from the injured side. f) Statistical analysis of the area of muscle fibers from the injured side quantified from Masson's trichrome staining images. g) Representative images of laser speckle imaging for nerve blood flow perfusion. h) Quantification of mean blood flow perfusion values. i) 3D weight‐bearing status of the rat toes in each group at 12 weeks after surgery. j) Statistical analysis of the sciatic function index (SFI) of the rats at 4, 6, 8, 10, and 12 weeks after surgery. *n* = 5 independent animals per group. Significantly different (one‐way analysis of variance (ANOVA)): ns, not significant, *p < 0.05, **p <0.01, and ***p < 0.001.

Next, electrophysiological assays were employed to test the conduction function of the regenerated sciatic nerve. External electrical stimulation was applied to induce muscle contraction, and compound muscle action potentials (cMAPs) were recorded using recording electrodes. The delay time of cMAPs was employed as an indirect indicator of myelination, where a longer delay time signifies slower nerve conduction. Additionally, the peak value of the action potentials reflects the functional reconstruction of the innervated muscle, with a higher peak indicative of enhanced muscle contraction ability.^[^ ^40^ ^]^ In the 12‐week electrophysiological evaluation, the cMAP peak amplitude of both the ANA and Mito‐ANA groups was less than that of the autograft group, but the cMAP of the Mito‐ANA group was markedly higher than that of the ANA group (**Figure** **7**a,b). There was no statistically significant difference in the delay time between Mito‐ANA and autograft groups, but both were higher than the ANA group (Figure 7c). The electrophysiological results indicate that Mito‐ANAs effectively restored the conduction ability of the regenerated sciatic nerve.

Representative macroscopic images of the separated gastrocnemius muscle from the injured and healthy sides show that the gastrocnemius muscle exhibited significant atrophy in the ANA group compared with Mito‐ANA and autograft groups (Figure 7d). Masson's trichrome staining of the gastrocnemius muscle revealed that the muscle fibers in autograft and Mito‐ANA groups had regular and intact contours, with slightly smaller fibers in the Mito‐ANA group compared with the autograft group. The ANA group showed denervated atrophy of the muscle fibers, with extensive infiltration of blue fibers (Figure 7d). To quantitatively evaluate muscle atrophy after denervation, the wet weight of the target muscle on the injured side was compared with that of the healthy side, and the recovery rate of the wet weight was calculated. The results showed that the wet weight ratio of the Mito‐ANA group was comparable to that of the autograft group and significantly higher than that of the control group (Figure 7e). The area of muscle fibers was calculated for three groups, with an average area of 889.2 ± 169.5 µm^2^ for the ANA group, 1257 ± 192.5 µm^2^ for the Mito‐ANA group, and 1489 ± 145.1 µm^2^ for the autograft group (Figure 7f). There was a significant difference between Mito‐ANA and ANA groups. The analysis of the target muscle‐related results suggests that the use of ANA alone leads to significant denervation atrophy of the target muscle, while Mito‐ANA transplantation promotes reinnervation of the target muscle after nerve injury.

The close relationship between the delivery of diverse nutrients to the site of injury and the capacity to expedite the repair process of surrounding nerves is well established. Therefore, reestablishing blood flow in PNI is also an indispensable factor in the repair process. Laser speckle imaging was used to evaluate the average blood perfusion capacity of the nerve. Both the Mito‐ANA and autograft groups exhibited higher intensity blood perfusion signals than those of the ANA group (Figure 7g). After 12 weeks postsurgery, the average blood perfusion results showed that ANA, Mito‐ANA, and autograft groups had average blood perfusion values of 300.3 ± 30.73, 388.2 ± 45.82, and 452.6 ± 16.45 PU, respectively (Figure 7h). This suggests that the use of Mito‐ANA enhances the blood perfusion capacity during nerve regeneration, and its therapeutic effect is superior to using ANA alone.

At 12 weeks postsurgery, gait analysis was performed on the three groups of experimental rats. 2D toe imprints and 3D force analysis showed that compared with the ANA group, the footprint of the injured side hind paw of the Mito‐ANA group was more regular, with a larger contact area and greater length and width. The toe pressure in the ANA group was concentrated on the sole, resulting in a lower footprint pressure (Figure 7i; Figure S8, Supporting Information). The sciatic functional index (SFI) results at five evaluation time points (4, 6, 8, 10, and 12 weeks) showed that there was no statistically significant difference between the Mito‐ANA and autograft groups, except for week 4. Starting from week 6, the therapeutic effect of Mito‐ANA became significantly superior to that of ANA. All three groups showed a recovery trend, with the autograft group demonstrating the overall best trend and fastest recovery, while the ANA group showed the poorest recovery. The Mito‐ANA group exhibited significant repair advantages (Figure 7j).

## 3 Discussion

ANA is considered a possible replacement for autologous nerve grafts for nerve defect repair, and the addition of cells and cellular derivatives is currently a preferred strategy to optimize their repair effect. Commonly used cell‐active additives include supporting cells, growth factors, and various extracellular vesicular components.^[^ ^41^ ^]^ Considering the high energy‐consuming of the nervous system and the pathological microenvironment of localized mitochondrial dysfunction accompanied by energy depletion after nerve injury,^[^ ^42^, ^43^ ^]^ we developed a new cell‐free therapy for ANA efficacy enhancement by transplanting hUCMSC mitochondria into the injured area for direct energy gifting.

First, hUCMSCs were lysed by a combination of mechanical and chemical methods, and high‐purity and functionally healthy mitochondria were prepared by multiple differential centrifugations, which were carried out at low temperatures and for the shortest duration possible. Given that mitochondria play a pivotal role as the powerhouse of the cell and impaired mitochondrial function can result in elevated levels of reactive oxygen species, the establishment of a standardized and efficient preparation protocol becomes imperative to ensure the stability of mitochondrial treatment. Additionally, quality control and characterization of mitochondria are essential to maintain consistency across subsequent interventions and guarantee their efficacy.^[^ ^44^ ^]^ We chose hUCMSCs as the donor of mitochondria due to their high proliferative capacity and relatively young cell state.^[^ ^45^ ^]^ It has been confirmed that mitochondria derived from young state cells have a superior ability to improve cellular function and reverse senescence.^[^ ^44^, ^46^, ^47^, ^48^ ^]^ hUCMSC‐Mitos were observed under TEM and the characteristic structures were clear and intact. Molecular biology methods were used to validate that the extracted products had high levels of expression of mitochondrial marker proteins COX IV and TOMM20, and low levels of cytoplasmic proteins, demonstrating that we extracted highly pure mitochondria. As mitochondria are subcellular organelles, the mitochondrial yield of cells varies according to strain and generation. Due to the dynamic regulation of fusion division in the mitochondria,^[^ ^49^ ^]^ it is difficult to quantify them by direct counting as in cells, and hence we used BCA protein quantification to quantify mitochondria indirectly for each acquisition.

SCs are key cells responsible for the formation of the myelin sheath around axons in the peripheral nervous system, and they have been the focus of research in peripheral nerve regeneration.^[^ ^50^ ^]^ Therefore, this study sought to investigate whether SCs can uptake exogenous mitochondria and explored the functional changes that occur after mitochondrial uptake. We obtained SCs according to a standardized SCs extraction, purification, and proliferation protocol and cocultured them with hUCMSC‐Mitos. It was discovered that similar to findings in other cell types such as cardiomyocytes,^[^ ^28^ ^]^ neurons,^[^ ^51^ ^]^ and immune cells,^[^ ^52^, ^53^ ^]^ SCs also exhibited an astonishing ability to rapidly uptake hUCMSC‐Mitos. The internalization process was activated within 10 min, and after 8 h, almost all SCs had taken up a substantial number of hUCMSC‐derived mitochondria. We observed that SCs internalized mitochondria on the first and third day, but on the fifth day, the fluorescence of mitochondria labeled with MitoTracker became weak and difficult to observe. However, as the limitation of MitoTracker which can only last for a few days, we were unable to confirm whether it was a quenching of the fluorescence or an inactivation of the mitochondria on the fifth day. Labeling mitochondria with lentiviral‐transfected cells may be a quality option because it avoids the potentially toxic effects of the chemical dyes and lasts for a longer period of time.^[^ ^54^ ^]^ Previous studies have mainly focused on an intriguing therapeutic mechanism involving MSCs transferring mitochondria to neighboring cells through the formation of tunneling nanotubes,^[^ ^55^ ^]^ which is seemingly facilitated by the pathological microenvironment following injury.^[^ ^56^ ^]^ By contrast, our in vitro coculture experiments demonstrated that normal SCs can also efficiently take up exogenous mitochondria directly through endocytosis.

In addition to ATP production through oxidative phosphorylation, mitochondria also have important regulatory functions in apoptosis, migration, differentiation, immune response, senescence, and autophagy.^[^ ^24^, ^57^ ^]^ Thus, it is essential to explore whether exogenous transplanted mitochondria can also play a regulatory role in recipient cells and determine the effective concentration at which mitochondrial action takes effect. We performed a proproliferation assay using CCK‐8 and explored the concentration of effect. We found that mitochondria had a proproliferative effect on SCs and their proliferative effect had a positive correlation with concentration and time within a certain range. Therefore, a concentration of 20 µg mL^−1^ of hUCMSC‐Mito intervention was used for subsequent in vitro experiments, and the third day after mitochondrial transplantation was the detection time point. The effectiveness of hUCMSC‐Mitos in promoting proliferation was further verified using the EdU cell proliferation assay. The wound‐healing assay revealed that hUCMSC‐Mitos promoted the migratory motility of SCs. Seahorse analysis is the gold standard for measuring cellular respiratory metabolism. We examined the bioenergetic profile of hUCMSC‐Mito‐treated SCs as well as blank‐treated SCs using the mitochondrial stress assay. Mitochondrial‐transplanted SCs exhibited higher levels of OCR in terms of basal respiration, maximal respiration, spare respiration, and ATP production compared with the control group, indicating that after mitochondrial transplantation, SCs had increased energy production and stress response capabilities. We speculate that the increase in energy may provide the foundation for SC proliferation and migration. Transcriptomic sequencing results were consistent with the in vitro experimental findings, which showed that hUCMSC‐Mitos enhanced SC functions, particularly in migration and motility. We discovered that hUCMSC‐Mitos upregulated angiogenesis and vascular morphogenesis in SCs. In subsequent in vivo experiments, we further investigated the microvascular formation and vascular remodeling during the regeneration process. KEGG pathway analysis showed that differential genes were enriched in the PI3K‐Akt signaling pathway, MAPK signaling pathway, and other pathways. Previous studies have demonstrated the mechanism of the PI3K‐Akt signaling pathway in promoting cell proliferation, migration, angiogenesis, and myelin formation.^[^ ^58^ ^]^

We obtained evidence of the positive regulatory effects of hUCMSC‐Mitos on SCs in vitro and then attempted to explore the effectiveness of hUCMSC‐Mitos in promoting nerve regeneration in vivo. ANA was prepared using a decellularization method and the removal of cellular components was confirmed through immunogenicity testing. The ANA preserved the 3D structure of native nerves, guiding axonal and vascular extension, and the presence of voids facilitated oxygen and mitochondrial accommodation. After transplanting the grafts into the sciatic nerve defects of rats, we tracked the fate of the mitochondria using in vivo animal imaging and immunofluorescence staining. It was found that 3 days after transplantation, the mitochondria aggregated at the ends of the nerve injury, perhaps because they were taken up by residual nerve tissue cells. The distribution area of fluorescence was larger at the proximal end than at the distal end, which may be related to the stronger metabolism of proximal SCs.^[^ ^38^ ^]^ Our study focused on SCs during the process of nerve regeneration and hUCMSC‐Mitos were internalized by SCs on both sides of the injury, with a higher fluorescence intensity and a larger area in the proximal SCs than in the distal ones, suggesting that the transplanted mitochondria primarily exert their effects in SCs at the proximal end of the injury site.

Moreover, we analyzed the regenerative microenvironment of nerves 3 days after Mito‐ANA transplantation and metabolomics analysis was performed to determine the metabolites and metabolic pathways. NAD+ was the main upregulated metabolite, which plays a crucial role in cellular energy metabolism and is essential for the maintenance of cellular function and the proper functioning of overall biological processes.^[^ ^59^ ^]^ The upregulation of metabolic pathways primarily involves the biosynthesis of pantothenic acid and CoA. These compounds play crucial roles in diverse metabolic reactions, such as the citric acid cycle and fatty acid biosynthesis.^[^ ^60^ ^]^ The KEGG regulatory network suggested an important role for metabolic networks associated with the TCA cycle—a pathway of sugar, lipid, and amino acid metabolism that plays an important role in regulating energy. Various nutrients can be biosynthesized through reversible reactions using common metabolic intermediates and all these processes require the TCA cycle as a mediator.^[^ ^61^ ^]^ The tissue Seahorse assay was used to resolve the energy production capacity of the damaged region after nerve injury. Results showed that both the respiratory and energy production capacities of Mito‐ANA were restored to a level comparable to that of autograft nerves but were significantly superior to that of the ANA group. We believe that Mito‐ANA significantly increased energy production by enhancing the TCA cycle and improved the regenerative microenvironment by empowering the injured region.

The evaluation of nerve regeneration in vivo showed that autograft nerves achieved the best regeneration outcomes, while Mito‐ANA demonstrated therapeutic efficacy comparable to them, better than ANA group. Based on immunofluorescence staining of NF200, S100β, CD31, and α‐SMA in newly formed nerve tissue sections at 3 and 12 weeks, Mito‐ANA showed superior ability to ANA in terms of nerve regeneration and angiogenesis. Electrophysiological tests showed that the recovery of nerve signal conduction in the Mito‐ANA group was comparable to that of the autograft group but superior to that of the ANA group, indicating a relatively high degree of remyelination. After nerve dissection, the target muscle lost its voluntary contraction function, muscle fibers underwent atrophy,^[^ ^62^ ^]^ and the affected toe had flexion contracture and toe width reduction. Throughout the process of nerve regeneration, reinnervation of the target muscles takes place, accompanied by a gradual enhancement in nutrient supply. These factors collectively contribute to the restoration of muscle fiber structure and a progressive normalization of footprints. Both Mito‐ANA and autograft demonstrated a more pronounced promotion of motor function recovery than ANA. Mito‐ANA also exhibited a significant improvement in blood perfusion compared with ANA, facilitating nutrient transport and oxygen supply during nerve regeneration.

To the best of our knowledge, this is the first study to promote injury repair by directly supplementing exogenous functional mitochondria to empower injured peripheral nerves. Our study provides evidence that the graft, which possesses energy‐giving capacity, can effectively facilitate axonal regeneration and microvascular revascularization. Nevertheless, the precise mechanisms underlying the effects of hUCMSC‐Mitos have yet to be fully elucidated. Additionally, although this study focused on SCs, the mitochondrial transfer behavior within the nerve regeneration microenvironment is not limited to SCs alone. The early mechanisms of hUCMSC‐Mitos in endothelial cells, fibroblasts, and immune cells may also play a role in the regenerative process. Further investigation of the ability and impact of mitochondrial uptake by different cells during nerve regeneration and a more comprehensive interpretation of the regulatory role of mitochondria in the complex microenvironment of peripheral nerves warrant further investigation. The efficiency of isolating mitochondria from cells is much greater than that of secreting mitochondrial vesicles, but naturally secreted mitochondrial vesicles have better stability and homing effects compared to purified isolated mitochondria.^[^ ^63^, ^64^ ^]^ Encapsulation of mitochondria with biomaterials may improve the stability of mitochondria in harsh pathological microenvironments. Moreover, encapsulating mitochondria with different types of cell membranes specific to different receptor cells may improve the homing effect of mitochondria to the respective receptor cells. Refinement of these works can provide a better theoretical basis for the clinical treatment of PNI using exogenous functional mitochondria.

## 4 Conclusion

In summary, hUCMSC‐Mitos were extracted and complexed with ANAs prepared by chemical decellularization to construct grafts with energy‐giving functions. The results of in vitro experiments showed that SCs efficiently internalized hUCMSC‐Mitos and exhibited enhanced functions in proliferation, migration, and energy production after hUCMSC‐Mito uptake. Mito‐ANA reshaped the pathological microenvironment in sciatic nerve‐injured rats, empowered the energy‐depleted microenvironment, significantly increased the rate of nerve regeneration, and promoted functional recovery compared with ANA without mitochondria. Overall, our findings suggest that the use of hUCMSC‐Mitos to enhance ANA may be a promising paradigm for PNI repair.

## 5 Experimental Section

### Extraction and Characterization of Mitochondria from hUCMSCs

Second‐generation research grade hUCMSCs (RC02003, Nuwacell) were purchased from Nuwacell Biotechnologies Co., Ltd. hUCMSCs were cultured to the third generation for mitochondrial extraction. Mitochondrial staining of hUCMSCs was performed using a solution containing 200 nm MitoTracker Red CMXRos (M7512, Invitrogen) in the medium. After incubation for 30 min, cells were washed with phosphate‐buffered saline (PBS) and collected. Mitochondrial extraction was performed using a mitochondria isolation kit (SM0020, Solarbio) and a Dounce homogenizer (2 mL capacity). Mitochondrial isolation was performed following the manufacturer's instructions: hUCMSCs were resuspended in 1 mL of Lysis Buffer (Solarbio) and homogenized 40–50 times in the Dounce homogenizer. The homogenate was transferred to a centrifuge tube, and after centrifugation at 1000 × *g* for 5 min, the supernatant was collected. This centrifugation step was repeated, and the supernatant was discarded after centrifugation at 12 000 × *g* for 10 min, resulting in a crude mitochondrial pellet. The pellet was resuspended in 0.5 mL of Wash Buffer, followed by centrifugation at 1000 × *g* for 5 min to collect the supernatant. After centrifugation at 12 000 × *g* for 10 min, the supernatant was removed and a highly purified mitochondrial pellet was obtained. The construction of purified mitochondria was observed using a transmission electron microscope (TEM), and the purity of purified mitochondria was assessed by Western blot analysis of mitochondrial marker proteins (TOMM20 and COX IV) and cytoplasmic marker proteins (β‐actin and GAPDH). The protein concentration of purified mitochondria was quantified using the BCA kit. The functional status of cells and mitochondria was measured using the ROS Assay Kit (S0033, Beyotime) and the ATP Assay Kit (S0027, Beyotime), and the procedure was carried out according to the instructions supplied by the manufacturer.

### Extraction, Purification, and Proliferation of Primary SCs

Four 3‐day‐old Sprague‐Dawley (SD) strain mice were euthanized, and their bilateral sciatic nerves were obtained after soaking in 75% ethanol for 20 min. The nerves were washed in Dulbecco's Modified Eagle Medium/Nutrient Mixture F‐12 (DMEM/F‐12) culture medium. A digestion solution consisting of trypsin/type I collagenase/DF‐12 (1:1:8) was prepared and used to digest the nerves at 37 °C for 30 min. After centrifugation, the supernatant was removed and nerve tissues were dissociated into a single‐cell suspension using 4 mL of SCs basal medium. The cells were then cultured for 24 h. Next, the medium was replaced with an SC purification medium and incubated for 72 h. The medium was then replaced with a complete medium. When the SCs reached an appropriate density, gentle digestion was performed with trypsin for cell passage and the expanded SCs were used for subsequent experiments.

### Observation of hUCMSC‐Mito Internalization in SCs

SCs (2 × 10^4^ cells per well) were seeded onto cell culture slides in a 24‐well plate. After 24 h, SCs were labeled with DiO (C1993S, Beyotime) to mark the cell membrane. Prestained hUCMSC‐Mitos were seeded in the same 24‐well plate and cocultured with SCs for 24 h. Then the SCs were washed with PBS and fixed with 4% paraformaldehyde (PFA), and the DNA of SCs was labeled with 4′,6‐diamidino‐2‐phenylindole (DAPI). Imaging was performed using a confocal panoramic scanner (Pannoramic, 3DHISTECH).

Live cell imaging was employed to record the real‐time uptake process of hUCMSC‐Mitos by SCs. SCs (5 × 10^4^ cells per well) were implanted onto a 6‐well plate for 24 h. Prestained hUCMSC‐Mitos were then seeded at a concentration of 20 µg mL^−1^ in the 6‐well plate. The culture plate was immediately placed in a high‐content analysis system (Operetta CLS, PerkinElmer). Cell behavior was recorded in Brightfield and Alexa 594 channels using Harmony software. The interval of the recorded parameter was set to 5 min, and continuous recording was performed for 12 h.

### Evaluation of SC Proliferation and Migration Behavior

SCs (3000 cells per well) were placed in a 96‐well plate, and proliferation capacity was evaluated using the CCK‐8 (CA1210; Solarbio). The experimental procedures were conducted following the manufacturer's instructions. Cells were incubated at 37 °C, and the culture supernate was replaced with a medium containing 10% CCK‐8 detection reagent (Solarbio) after 24 and 72 h, respectively, and incubated for 2 h. The absorbance of the supernatant was detected at 450 nm using a microplate spectrophotometer (Take3, Epoch).

The EdU assay was performed using the Cell‐Light Apollo Stain Kit (C10310, Ribobio) following the manufacturer's instructions. SCs (2 × 10^4^ cells per well) were placed in a 24‐well plate and cocultured with hUCMSC‐Mitos for 72 h. Afterward, SCs were incubated with 50 µm of EdU medium for 2 h. SCs were fixed with 4% PFA and permeabilized with 0.5% Triton X‐100. Apollo reaction cocktail was added and cells were incubated for 30 min. DNA was stained with Hoechst 33342 and observed under fluorescence microscopy (Nikon, Tokyo). The EdU incorporation rate is the proportion of EdU‐positive SCs in the total number of SCs in the field of view.

Cell migration ability was assessed using the wound healing assay. SCs (2 × 105 cells per well) were seeded in a 6‐well plate. After 12 h of cultivation, a sterile 200 µL pipette tip was used to create a scratch on the bottom of the culture plate. SCs were washed with PBS to remove any floating cells and then placed in a cell culture incubator for 3 days. The wound healing process was meticulously monitored using ImageJ software, which facilitated the measurement of the ratio between the healed area and the initial gap area. This analytical approach enabled the assessment of wound closure and repair dynamics.

### SC Bioenergetics Profiling Assay

The Cell Mito Stress Test (CMST, 103591‐100, Agilent) was performed using the Seahorse XF96 Analyzer. The experimental conditions were selected based on the recommendations in the user manual. The hUCMSC‐Mito group and the control group of SCs (2 × 10^4^ cells per well) were seeded onto a microplate (Agilent). The plate was then incubated overnight in a cell culture incubator, and the media were replaced with DMEM (Agilent). The microplate with hydrated sensor cartridges was placed in a non‐CO_2_ incubator for 1 h before measurement. In the CMST, 1.5 µm of oligomycin (Agilent) was added to well A of the drug plate, 0.5 µm of FCCP (Agilent) to well B, and 0.5 µm of Rot/AA (Agilent) to well C. The microplate with hydrated sensor cartridges was then loaded onto the instrument, and the program was initiated to run the measurement. The Report Generator (Agilent) automatically calculated the CMST parameters of the cells, including basal respiration, maximal respiration, spare respiratory capacity, and ATP production.

### Transcriptomic Analysis

SCs (5000 cells cm^−2^) were seeded in culture dishes under the conditions of hUCMSC‐Mitos addition and blank control and cultured for 3 days. Each group had three biological replicates, with 1 × 10^6^ cells per sample. SCs were lysed with TRIzol (15596018, Life Technologies) for 5 min. Library construction and transcriptome data analysis were performed by Novogene Co. Ltd. (Beijing, China). Quality control was conducted using the Agilent 2100 Bioanalyzer, followed by Illumina NovaSeq 6000 sequencing. GO was divided into three modules: CC, MF, and BP. After RNA sequencing, GO and KEGG pathway enrichment analyses of DEGs were performed using the clusterProfiler (3.8.1).

### Construction and Characterization of ANAs and Mito‐ANAs

Ten SD rats were used. Bilateral sciatic nerves were dissected, with a length of 15 mm. The nerves were immersed in physiological saline for 12 h, washed in PBS, and immersed in physiological saline for a further 12 h at −80 °C. After thawing, the nerves were washed with PBS and then placed on a shaker at 60 rpm in 1% sodium deoxycholate (Sigma, USA) for 1 day. Samples were washed until no foam was generated. Subsequently, ANAs were sealed and stored at −20 °C after Cobalt‐60 irradiation. Approximately 30 µL of mitochondrial suspension was evenly injected along the long axis of the ANA using a 31G insulin syringe needle, insulin syringes were pushed using a constant speed syringe push pump to fill the lumen of the ANA tubing with the mitochondrial suspension. The effectiveness of cellular removal in the ANA compared with natural nerves and the loading of mitochondria on the ANA were assessed using DNA quantification and immunofluorescence staining methods, followed by observation under a scanning electron microscope.

### In Vivo Assays: Surgical Procedure

All animal experiments were approved by the Experimental Animal Ethics Committee of PLA General Hospital (approval number: 2016‐x9‐07). Female SD rats were randomly divided into three groups (*n* = 12 rats per group): ANA, Mito‐ANA, and AUTO. The rats were weighed and then anesthetized with 30 mg kg^−1^ pentobarbital sodium via abdominal cavity injection. Under aseptic conditions, the right sciatic nerve was cut off to create a 10 mm sciatic nerve defect, and the graft was sutured using 10‐0 tension‐free band sutures. After achieving adequate hemostasis, the operative wound was closed using 4‐0 sutures, and the incision was disinfected with iodine. The animals were provided with food and water and subjected to a 12 h light/dark cycle. Routine blood tests were used for immune and inflammatory markers.

### Mitochondrial Fate Tracking

I) Mito‐ANA was prepared using hUCMSC‐Mitos prestained with MitoTracker Red. ANA (with the addition of a medium instead of hUCMSC‐Mitos) was transplanted into the nerves as a negative control to establish the model. Rats were anesthetized on days 1 and 3 after surgery and placed in an IVIS Spectrum (PerkinElmer, Waltham, MA) imaging system to observe the fluorescence signal distribution of MitoTracker Red. II) On day 3, rat nerve grafts were fixed and dehydrated for frozen sectioning. Combined staining of S100β, MitoTracker Red, and DAPI was performed to detect the cellular uptake of hUCMSC‐Mitos within the graft.

### Energy Metabolism Microenvironment Analysis

I) Metabolomics: A nontargeted metabolomics analysis of the nerves was carried out in ANA and Mito‐ANA groups after three days following surgery using liquid chromatography and mass spectrometry techniques. The obtained results were then subjected to multivariate statistical analysis, specifically partial least‐squares discriminant analysis, to identify and explore the differences between the two groups. Additionally, hierarchical clustering analysis and metabolite correlation analysis were employed to investigate the relationships among different metabolites. Functional analysis of the identified metabolites was performed to determine their significance and potential roles in the observed differences. Data analysis for the metabolomics study was conducted by Novogene Co. Ltd. (Beijing, China). II) Bioenergetics profiling: To study the bioenergetics profile of neural tissues transplanted with ANA and Mito‐ANA after 3 days, fresh tissue slices of the proximal sciatic nerve were prepared using a vibrating microtome (Leica VT1200S). Samples were cut to a thickness of 250 µm and then seeded onto a microplate (Agilent). Subsequent steps followed the same procedure as that of the CMST of SCs described above.

### Neural Tissue Sectioning

At weeks three and 12 after the surgery, regenerated nerve tissues from the injured side of the rats in the three groups were collected following anesthesia. The collected tissues were promptly immersed in a 4% PFA solution and fixed for 24 h. After fixation, samples were dehydrated using a sucrose solution and subsequently embedded in an optimal cutting temperature compound (OCT compound). The embedded samples were then sliced into 12 µm thick sections using a cryostat. For the tissues collected at week three, longitudinal sectioning was performed, while transverse sectioning was employed for the tissues collected at week 12.

### Electrophysiological Testing

At 12 weeks after surgery, the animals were anesthetized and the injured side sciatic nerve was exposed. A multichannel physiological signal acquisition and processing system (RM6240EC, Chengyi) was utilized for testing. The stimulating electrode was positioned near the proximal end of the graft, while the recording electrode was placed parallel to the gastrocnemius muscle. The stimulation current was set at 0.2 mA, and cMAPs were elicited and recorded. The latency time and peak values of cMAP in the muscle were analyzed for each rat in each experimental group.

### Wet Weight Measurement of Gastrocnemius

At 12 weeks after surgery, the rats were anesthetized and bilateral gastrocnemius muscles were promptly weighed. The wet weight ratio of the injured side muscle to the contralateral side muscle was determined by dividing the muscle mass of the injured side by the muscle mass of the contralateral side.

### Assessment of Blood Perfusion

At 12 weeks postoperation, the affected side sciatic nerve in rats was detected under anesthesia using Laser Speckle Contrast Imaging (LSCI, RFLSI Pro, RWD). Changes in perfusion over time were analyzed using LSCI review software (RWD). Regions of interest were selected based on the degree of neural blood flow enhancement for measurement.

### Gait Analysis

At weeks 4, 6, 8, 10, and 12 after surgery, footprints were collected using an animal gait analysis system (CatWalk, Noldus) and recorded with a camera to capture the walking trajectory. CatWalk XT 10.6 software (Noldus) was used to calculate the SFI. Three footprints were measured for both the injured side (E) and the normal side (N). The parameters measured included the toe width (TS), intermediate toe width (ITS), and print length (PL). The SFI was calculated using the following formula: SFI = 109.5 (ETS − NTS)/NTS − 38.3 (EPL − NPL)/NPL + 13.3 (EIT − NIT)/NIT − 8.8.

### Histological Staining of Regenerated Neural Tissues: Immunofluorescence Staining

Samples were fixed in 4% PFA and then washed with PBS, followed by incubation with goat serum for 30 min. After blocking with goat serum, samples were incubated with primary antibodies at 4 °C overnight. The primary antibodies used included rabbit anti‐S100β (ab52642, Abcam), mouse anti‐NF200 (N0142, Sigma), rabbit anti‐CD31 (ab222783, Abcam), and mouse anti‐ɑ‐SMA (ab7817, Abcam). Subsequently, samples were washed and the slices were incubated with secondary antibodies at room temperature for 2 h in the dark. The secondary antibodies used included goat anti‐mouse Alexa Fluor488 (ab150117, Abcam), goat anti‐rabbit Alexa Fluor594 (ab150080, Abcam), and goat anti‐rabbit Alexa Fluor488 (ab150077, Abcam). DNA staining was performed using 4′,6‐diamidino‐2‐phenylindole (AR1176, BOSTER) for 10 min. Samples were then imaged using a confocal panoramic scanner (Pannoramic, 3DHISTECH).

### Toluidine Blue Staining and TEM

At 12 weeks postsurgery, nerve samples were fixed using a 2.5% glutaraldehyde solution and 1% osmium tetroxide solution, followed by dehydration and embedding. Transverse sections of 2.5 µm thickness were obtained for toluidine blue staining, and imaging was performed using a confocal panoramic scanner (Pannoramic, 3DHISTECH). Cross‐sectional ultrathin slices were stained with lead citrate and uranyl acetate and observed using a TEM. The myelinated nerve fiber density was quantified from the toluidine blue images using Image‐Pro Plus 6.0. Additionally, the nerve fiber diameter and average G‐ratio were quantified from TEM images.

### Masson's Trichrome Staining

The gastrocnemius muscle was fixed in 4% PFA, followed by dehydration in a graded ethanol series. Subsequently, the muscle was embedded in paraffin blocks. Transverse sections of 5 µm thickness were prepared from the embedded blocks. Masson's trichrome staining—a histological technique used to visualize collagen fibers and muscle tissue—was performed on these sections. Imaging of the stained sections was carried out using a confocal panoramic scanner (Pannoramic, 3DHISTECH). The acquired images were then used for the analysis of the cross‐sectional area of the gastrocnemius muscle fibers.

### Statistical Analysis

All experiments were conducted with a minimum of three replicates to ensure statistical robustness. Image Pro Plus 6.0 software was used to process and analyze the acquired images. Data analysis and graph plotting were performed using GraphPad Prism 7.0 software. Quantitative data were presented as mean ± standard deviation (X ± s), representing the central tendency and dispersion of the data, respectively. Comparisons of differences of means among multiple groups were assessed using one‐way analysis of variance. Pairwise comparisons were performed using Student's *t*‐test. A *p*‐value of ≤0.05 was considered statistically significant.

## Conflict of Interest

The authors declare no conflict of interest.

## Supporting information

Supporting Information

Supplemental Movie 1

## Data Availability

The data that support the findings of this study are available from the corresponding author upon reasonable request.
