# Supplementary material for: Mesenchymal Stem Cell‐Derived Mitochondria Enhance Extracellular Matrix‐Derived Grafts for the Repair of Nerve Defect
Source: Adv Healthc Mater. 2023 Nov 16;13(3):2302128. doi: 10.1002/adhm.202302128 (PMC11468562; doi:10.1002/adhm.202302128)
Supplement: Supplementary file 1 — Supporting Information [file ADHM-13-2302128-s002.pdf]

ADVANCED HEALTHCARE MATERIALS

Supporting Information

for *Adv. Healthcare Mater.*, DOI 10.1002/adhm.202302128

Mesenchymal Stem Cell-Derived Mitochondria Enhance Extracellular Matrix-Derived Grafts for the Repair of Nerve Defect

Jun Bai, Bingbing Yu, Chaochao Li, Haofeng Cheng, Yanjun Guan, Zhiqi Ren, Tieyuan Zhang, Xiangyu Song, Zhibo Jia, Tianqi Su, Benzhang Tao, Haihao Gao, Boyao Yang, Lijing Liang, Xing Xiong, Xingyu Zhou, Lan Yin, Jiang Peng, Aijia Shang* and Yu Wang**

Supplementary information

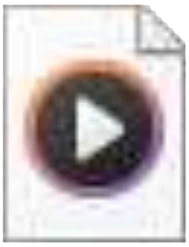

SCs internalized hUCMSC-Mitos.w

Mov.S1. Dynamic process of SCs internalizing hUCMSC-Mitos. (The cell contour was observed under bright field and mitochondria were labeled with red fluorescent dye.)

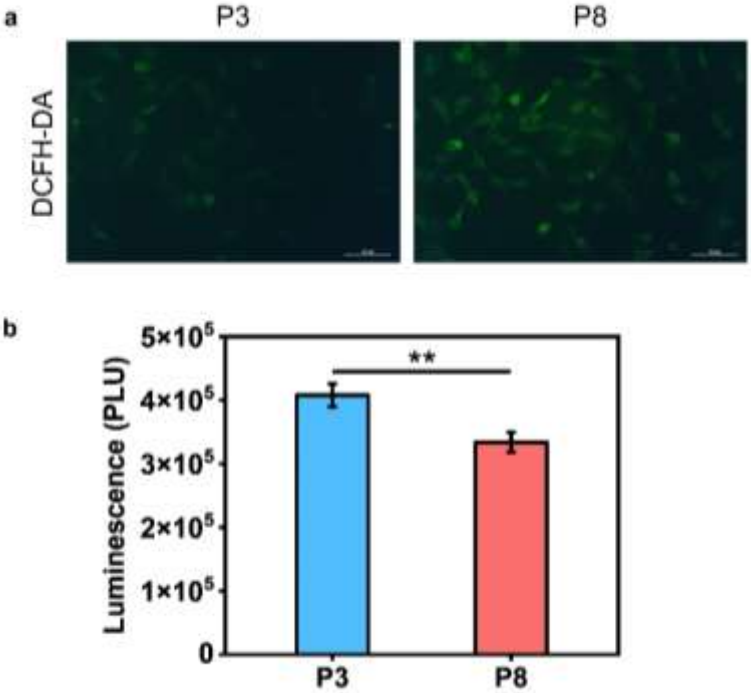

Fig. S1. Comparison of mitochondrial function in low-passage (P3) hUCMSCs and high-passage (P8) hUCMSCs. (a) ROS staining of hUCMSCs (ROS: DCFH-DA) (b) Detection of ATP content within hUCMSCs. Significantly different (one-way analysis of variance [ANOVA]): **p <0.01.

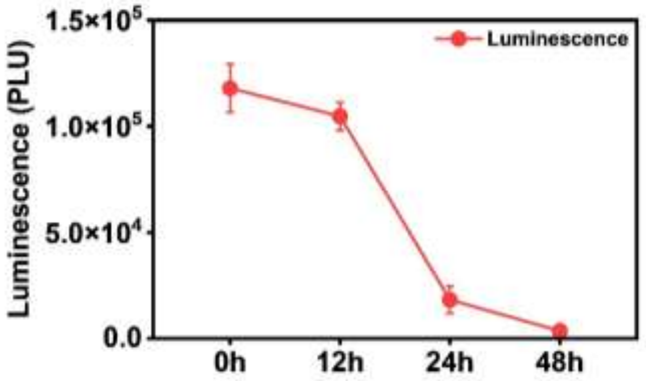

Fig. S2. Quantification of ATP on extracellular mitochondria. (The detection time points were 0 h, 12 h, 24 h and 48 h.)

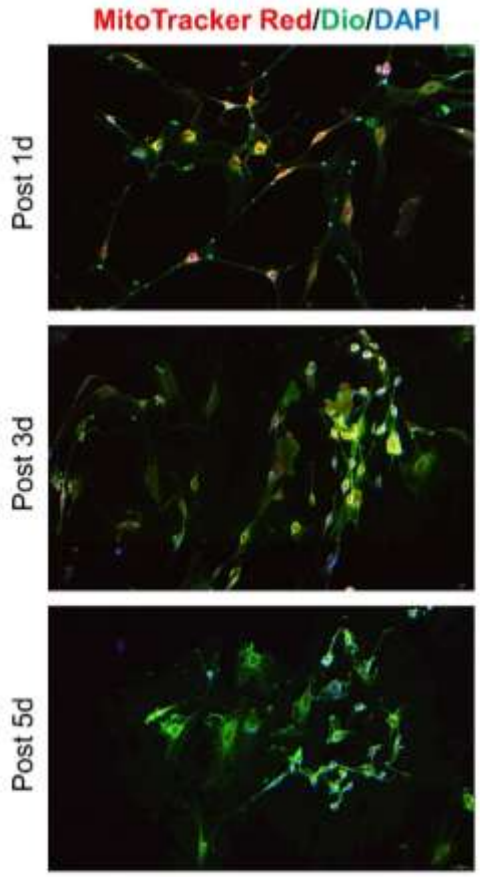

Fig. S3. Uptake of hUCMSC-Mitos by SCs (Cell membrane: DiO; hUCMSC-Mitos: MitoTracker Red; Nucleus: DAPI). (The detection time points were day 1, day 3 and day 5.)

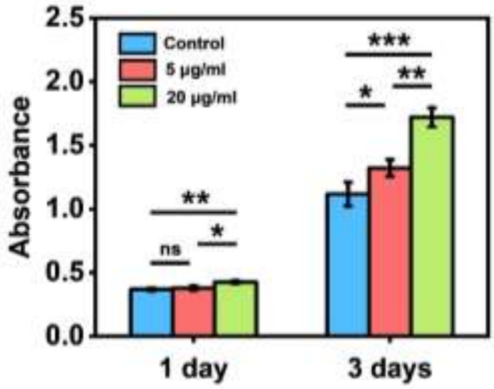

Fig. S4. CCK8 cell proliferation assay. (The detection time points were day 1 and day 3, and the concentrations of mitochondria were high and low.) Significantly different (one-way analysis of variance [ANOVA]): ns, not significant, *p < 0.05, **p <0.01 and ***p < 0.001.

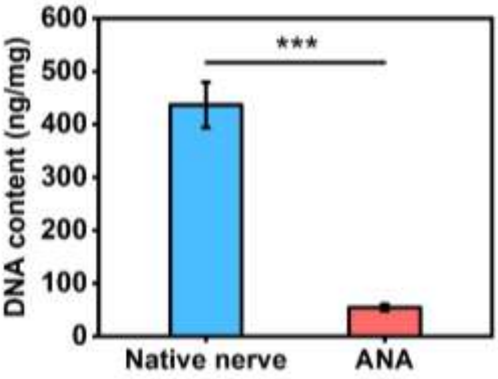

Fig. S5. DNA content of native nerve and ANA. Significantly different (one-way analysis of variance [ANOVA]): ***p <0.001.

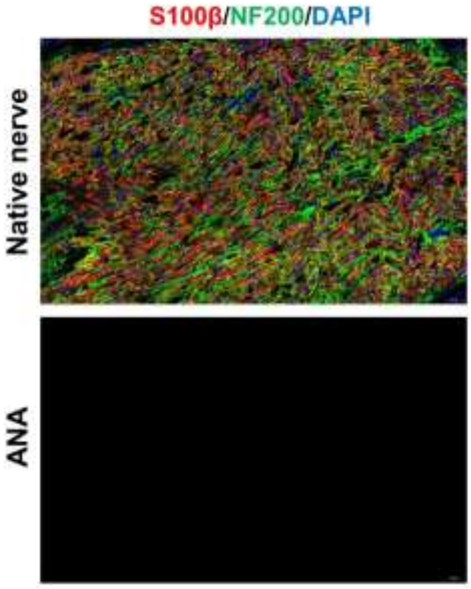

Fig. S6. Antigen detection of native nerve and ANA. (SCs: S100-β; Axons: NF200; DNA: DAPI)

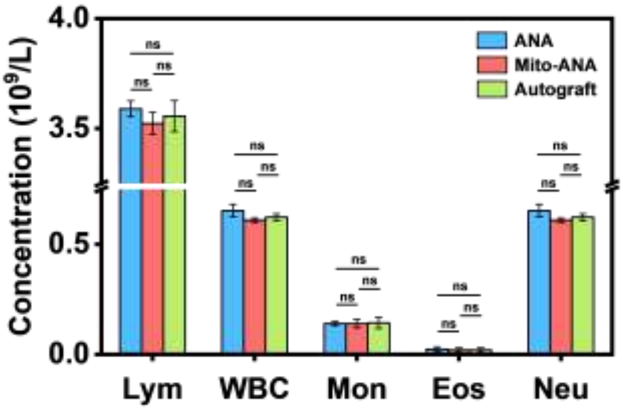

Fig. S7. Routine blood tests for inflammation and immune response after graft implantation. Lym: lymphocytes ($\times 10^9$ L^{-1}); WBC: white blood cell ($\times 10^9$ L^{-1}); Mon: monocytes ($\times 10^9$ L^{-1}); Eos: eosinophils ($\times 10^9$ L^{-1}); Neu: neutrophils ($\times 10^9$ L^{-1}). Significantly different (one-way analysis of variance [ANOVA]): ns, not significant.

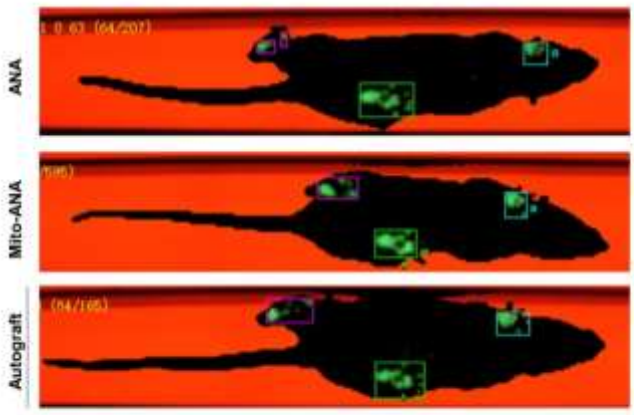

Fig. S8. Representative 2D plantar profile of gait. (The inside outline of the purple frame is the injured side foot.)
